# Technical Development of a New Semispherical Radiofrequency Bipolar Device (RONJA): *Ex Vivo* and *In Vivo* Studies

**DOI:** 10.1155/2014/532792

**Published:** 2014-04-09

**Authors:** Petr Vavra, Marek Penhaker, Jan Grepl, Jana Jurcikova, Jiri Palecek, Michal Crha, Jana Nowakova, Martin Hasal, Martina Skrobankova, Petr Ostruszka, Peter Ihnat, Patricie Delongova, Dana Salounova, Nagy A. Habib, Pavel Zonca

**Affiliations:** ^1^Department of Surgery, University Hospital Ostrava, 17. listopadu 1790, 708 52 Ostrava-Poruba, Czech Republic; ^2^Department of Cybernetics and Biomedical Engineering, Faculty of Electrical Engineering and Computer Science, VSB-Technical University of Ostrava, 17. listopadu 15, 708 00 Ostrava-Poruba, Czech Republic; ^3^Department of Surgery, Faculty of Medicine, University of Ostrava, Syllabova 19, 703 00 Ostrava 3, Czech Republic; ^4^Liver and Pancreas Surgery, Hammersmith Hospital, Imperial College London, Du Cane Road, London W12 0HS, UK; ^5^Faculty of Mechanical Engineering, VSB-Technical University of Ostrava, 17. listopadu 15, 708 00 Ostrava-Poruba, Czech Republic; ^6^Department of Vice-President for Science and Research, University Hospital Ostrava, 17. listopadu 1790, 708 52 Ostrava-Poruba, Czech Republic; ^7^CEITEC VFU, University of Veterinary and Pharmaceutical Sciences Brno, Palackého 1/3, 612 42 Brno-Královo Pole, Czech Republic; ^8^Institute of Pathology, University Hospital Ostrava, 17. listopadu 1790, 708 52 Ostrava-Poruba, Czech Republic; ^9^Faculty of Medicine, University of Ostrava, Syllabova 19, 703 00 Ostrava 3, Czech Republic; ^10^Department of Mathematical Methods in Economy, Faculty of Economics, VSB-Technical University of Ostrava, 17. listopadu 15, 708 00 Ostrava-Poruba, Czech Republic

## Abstract

The aim of this study is to inform about the development of a new semispherical surgical instrument for the bipolar multielectrode radiofrequency liver ablation. Present tools are universal; however they have several disadvantages such as ablation of healthy tissue, numerous needle punctures, and, therefore, longer operating procedure. Our newly designed and tested semispherical surgical tool can solve some of these disadvantages. By conducting an *in vivo* study on a set of 12 pigs, randomly divided into two groups, we have compared efficiency of the newly developed instrument with the commonly used device. Statistical analysis showed that there were no significant differences between the groups. On average, the tested instrument RONJA had shorter ablation time in both liver lobes and reduced the total operating time. The depth of the thermal alteration was on average 4 mm larger using the newly tested instrument. The new radiofrequency method described in this study could be used in open liver surgery for the treatment of small liver malignancies (up to 2 cm) in a single application with the aim of saving healthy liver parenchyma. Further experimental studies are needed to confirm these results before clinical application of the method in the treatment of human liver malignancies.

## 1. Introduction

Hepatocellular carcinoma (HCC) is the sixth most common cancer, representing 6% of all cancers, the third most common cause of cancer death, and the most common primary malignancy of the liver [1]. Most HCCs are developed in patients with risk factors such as chronic hepatitis B or C and nonviral liver cirrhosis, which may be associated with alcoholic liver disease or nonalcoholic fatty liver disease [2]. It is estimated that 15–20% of colorectal cancer patients present synchronous liver metastases. Moreover, liver metastases will develop in half of patients with colorectal cancer in the course of their disease [3].

Metastases of colorectal carcinoma (CLM) and hepatocellular carcinoma (HCC) are the most frequent indications for liver surgery.

Liver resection is the first-line treatment option for patients with primary and secondary liver tumors and well-preserved liver function. A better liver function assessment, increased understanding of segmental liver anatomy, using more accurate imaging studies and the technical progress in surgery are the most important factors that have led to reduced mortality and morbidity of liver tumors [4].

Radiofrequency energy has been used in medicine for more than one hundred years and was gradually incorporated into liver surgery. Radiofrequency ablation was a promising method that gradually developed, becoming one of the standard methods of the treatment of primary and secondary liver malignancies [5–8].

## 2. Materials and Methods

For creating the prototype, a 3D model of the instrument had to be prepared. The prototype was produced by 3D printing, which is more accurate. The equipment needed for its production was provided by the Faculty of Metallurgy and Materials Engineering of the Technical University Ostrava. The practical test was then performed at the University Hospital Ostrava using a standard radiofrequency generator.

### 2.1. RONJA Device Description

**Figure 1:**
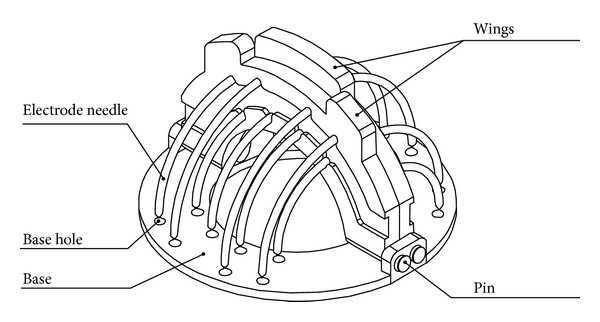
RONJA—new radiofrequency surgical tool.

A new layout of electrodes contains three and two groups of electrodes in a square shape positioned on the edges of a regular polygon. Three groups of electrodes are positioned on one electrode wing and the other two groups are positioned on the second wing (Figure 1). Electrode needles on each wing burn a semispherical area in the liver tissue. The second wing burns an area between the burned areas by the first wing. It is necessary to turn the instrument by 90° between each step and to burn the tissue in four steps.

### 2.2. RONJA Device Design and Construction

**Figure 2:**
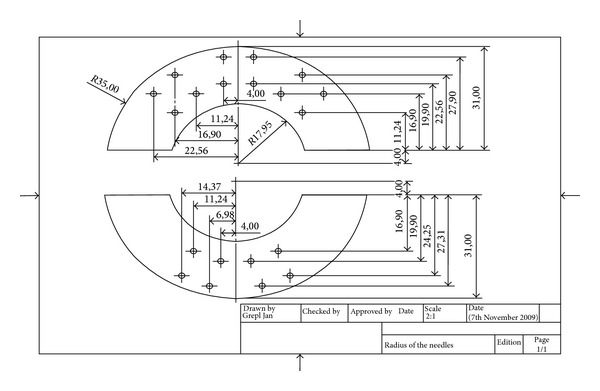
Scheme of a new electrodes layout of our radiofrequency tool RONJA.

**Figure 3:**
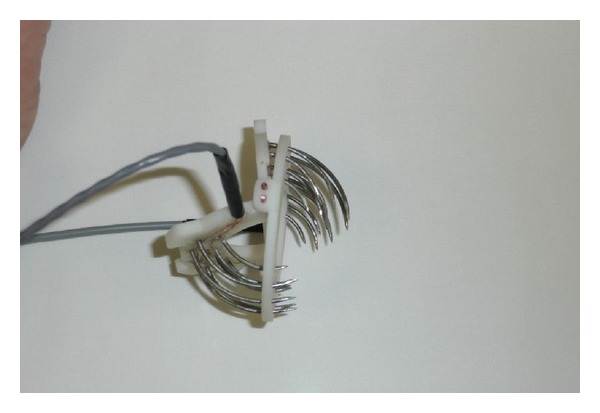
Prototype of the semispherical tool for* ex vivo* study.

**Figure 4:**
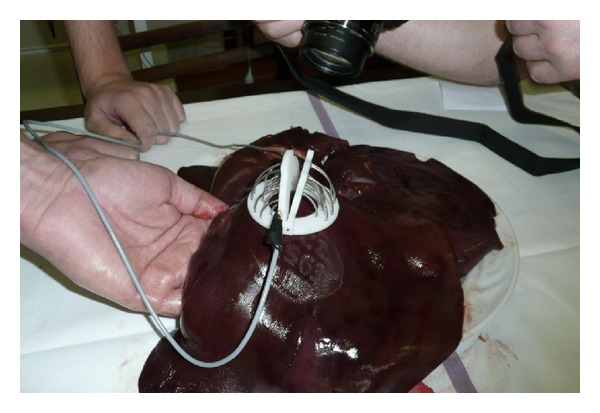
Application of RONJA device—*ex vivo* experiment.

Both wings and ring base of this new surgical instrument were created with the rapid prototyping technology on dimension SST 768 directly from the 3D CAD model. This machine uses FDM (fused deposition modeling) technology to create components in a very short time from ABS plastic material. Stainless steel was used to produce the electrode needles (Figure 3). The new instrument RONJA, which has an asymmetrical electrode layout, consists of a ring-shaped base, two wings with fixed electrode needles, and two connecting power wires. In the base ring, there are a number of holes to guide the electrode needles into the tissue (Figure 2). The electrode needles with a quarter-circle shape are bipolar. The needles are powered in each wing separately, between the inner and outer semicircles. One pole of the RONJA instrument is made by one semicircle of needles. Both wings are connected to the base through pins in such a way that, after insertion of both wings with needles into the base, a half-hemisphere shape of necrotic tissue will be created around the tumor (Figure 4).

The idea of constructing this tool with an asymmetrical layout of electrodes is completely new and it has not been used for these types of instruments so far.

### 2.3. *Ex Vivo* Testing

*Ex vivo* testing took place at the University Hospital Ostrava on porcine liver tissue because of its similarity to human liver.

**Figure 5:**
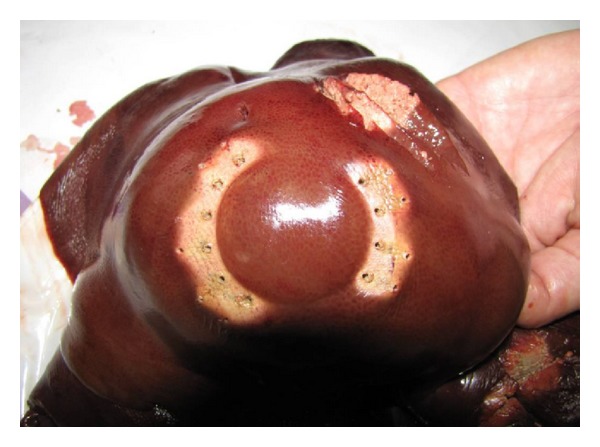
Result of radiofrequency ablation—semispherical necrosis of liver tissue.

The main goal of the testing was to understand whether the new proposed electrode layout is suitable for sufficient tissue burning and for creating a safety zone around the tumor (Figure 5). Another goal of the study was to discover any potential faults and to determine power level for* in vivo *testing. The instrument was connected to a commonly used radiofrequency generator. This generator is used in human medicine for surgery with currently available instruments.

### 2.4. *In Vivo* Testing

*In vivo* testing was conducted in 2012 on a set of 12 pigs (*Sus scrofa domestica*) randomly divided into two groups. The experiment was divided into three phases. In the first phase, the animals in both groups underwent middle laparotomy and radiofrequency-assisted liver resection under general anesthesia. Pigs in group A underwent liver resection using the new tested tool RONJA and pigs in control B group were operated with a commonly used four-electrode bipolar resection device. The next phase of the study followed 14 days after the primary operation and was focused on inspectional laparoscopy. In the last phase, 30 days after the first intervention, all the pigs were euthanized and samples were pathologically evaluated.

The experimental project (no. 65/2012) was approved by the expert committee on animal welfare of the Veterinary and Pharmaceutical University Brno according to the law on the protection of animals against cruelty, as amended by paragraph 18, Act no. 246/1992 Coll. The experiment was approved by the Ministry of Education, Youth and Sport of the Czech Republic and was granted permission to be carried out on the 7th of September 2012. Pigs, aged one year, were provided by the Research Institute of Animal Production in Uhrineves, Department of Pig Breeding in Kostelec. Address: Komenskeho 1240, 51741, Kostelec, Czech Republic, accreditation number 444/2011-MZE-17214, valid until April 6, 2016).

All three phases of the* in vivo *experiment took place in the research operating theatre of the Faculty of Veterinary Medicine of the Veterinary and Pharmaceutical University Brno. The surgical team consisted of two surgeons from the surgical department of the University Hospital Ostrava as well as two veterinary surgeons from the Veterinary and Pharmaceutical University Brno. Prior to the first surgery, every pig underwent an examination of its blood count, biochemistry, and hemocoagulation. All pigs were intramuscularly premedicated. Anesthesia was induced by intravenously administered Propofol (Norofol, Norbrook Lab. Ltd., North Ireland) (0.5–1 mg/kg) and then maintained by constant rate infusion of Propofol (0.1 mg/kg/min). All the instruments used for the middle laparotomy were supplied by B. Braun Medical, AESCULAP division. Monopolar electrocoagulation EMED ES 350 with the output of 255 W was used for this performance and a laparotomy retractor was applied. Two liver resections were performed on each pig: one from the right lateral lobe and one from the left lateral lobe of the liver. The liver resection was carried out using radiofrequency-assisted resection technique. The subsequent liver transection was performed using a scalpel or surgical scissors. Minimal bleeding (not more than 20 mL) from the resection line was treated with the radiofrequency instrument as well. Resected samples were fixed in 10% formalin solution for subsequent histopathological examination. After inspection of the surgical field, the abdominal laparotomy was closed in layers. Special antiseptic spray, silver aluminum aerosol and bandage protection against contamination (Henry Schein, Inc., Germany), was applied on the surgical wound. The pigs, under the control of a veterinary anesthesiologist, were recovered from general anesthesia and housed in standard breeding boxes. Postoperatively, the animals were given amoxicillin (15 mg/kg) and meloxicam (0.4 mg/kg) intramuscularly.

14 days after the liver resection, the second phase was executed to determine the status of healing or postoperative complications in the abdominal cavity of each animal. For this purpose, a diagnostic laparoscopy of each pig was performed to assess the postoperative findings. AESCULAP (B. Braun Medical., s.r.o., the Czech Republic) laparoscopy instrumentation was used for surgery: a human version of the endoscopic video unit TELE PACK X and laparoscopic optics HOPKINS with the outer diameter of 5 mm and the length of 290 mm (KARL STORZ Endoscopy, Germany).

The last phase of the testing was performed 30 days after the first surgery and autopsies of the 12 pigs were performed by a veterinary pathologist from the Institute of Pathological Morphology and Parasitology of the Veterinary and Pharmaceutical University Brno in the presence of a pathologist from the University Hospital Ostrava. Slaughtering was done by the protocol,* lege artis*. The animal carcasses were disposed by a rendering service. All animals were opened in abdominal cavity, then in thoracic cavity, followed by removal of the organs.

### 2.5. Statistical Analysis

Data between the standard device group and RONJA group were compared using the median test for continuous variables and Fisher's exact test for categorical variables. Data are presented as mean ± standard error of mean (S.E.M.) and median for continuous variables and as counts and percentages for categorical variables (complications). Statistical analysis was performed with IBM SPSS software version 18.0. A two-sided value of *P* < 0.05 was considered statistically significant for all analyses.

## 3. Results and Discussion

### 3.1. *Ex Vivo* Test of the New Layout

The radiofrequency generator was set to 125 W output for the first test. Figure 5 shows burned tissue after insertion and ablation by both electrode wings. Areas were burned perfectly even between the square needle groups. With this configuration, only two steps would be potentially required. Time required to burn the tissue in first test was 43 sec.

**Figure 6:**
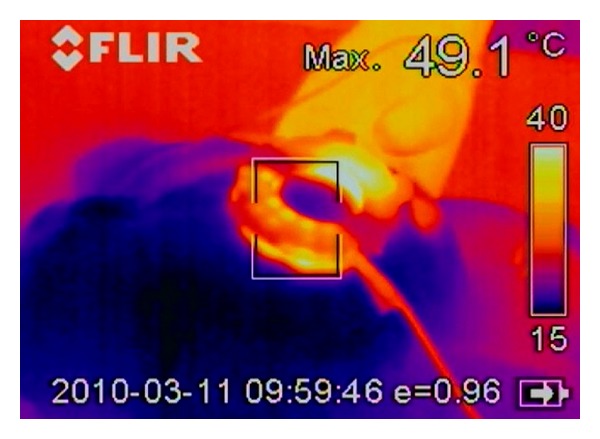
Thermal camera image of the resection of liver parenchyma.

In the second test, the radiofrequency generator was set to lower power output, default 90 W. The tissue was burned only in square groups of electrodes. Four steps were necessary to create a closed safety zone. The first and second steps took 37 sec and the third and fourth ones 9 sec. The thermal imaging from infrared camera showed that the maximum temperature around 64°C was reached in the liver tissue and on needles during the procedure (Figure 6). Maximum temperature on the instrument surface was 33°C, meaning that use of this instrument is absolutely safe for the surgeon.

*Ex vivo *trial helped us establish the power level for* in vivo* testing.

### 3.2. *In Vivo* Test of RONJA Tool

For comparison and statistical evaluation,* in vivo* testing data was collected including results of blood samples, the values recorded during surgery, and also the histopathological evaluation. The overall blood analysis showed no marked differences in the observed values before resection, on the 14th day after surgery, or on the 30th postoperative day.

During liver surgery, the total operating time and ablation time of lobes were measured and compared in both groups (Table 1).

The tested instrument RONJA had shorter ablation time in left lobe by 2.84 minutes and 1.17 minutes in right lobe than the commonly used device. The newly designed instrument also shortened operating time by 11.5 minutes compared to the standard device (Table 1). Statistical analysis using the median test (*P* value was computed by Fisher's exact test) failed to demonstrate any significant differences between the two groups. The power delivery of the radiofrequency energy generator was optimized during the surgical procedure. The power supply was reduced to 80 W after observing it had the same effect as the supply of 90 W. The commonly used device had a power supply of 90 W. Ablation times were determined automatically by the radiofrequency energy generator (Table 1).

Only one complication occurred during the liver surgery (Table 2). A spleen was deserosated in a pig in tested group A. In this animal, a serous effusion of 250 mL was observed during pathomorphological evaluation.

The control laparoscopy showed postoperative adhesions in all 12 animals. No serious postoperative complications occurred. Only one adverse event appeared during an inspection laparoscopy. The transverse colon of one pig in experimental group A was injured when establishing capnoperitoneum. There were no liver abscesses, biliary leaks, subhepatic abscesses, hematomas, or signs of peritonitis in any animal of both groups.

Death of one pig in control group B occurred in the postoperative period after laparoscopy performance. An autopsy was executed and an acute catarrhal-purulent bronchopneumonia, tonsillitis, and rhinitis were detected. The cause of death was determined as animal circulatory failure due to respiratory insufficiency in the postoperative period in connection to mycoplasmal infection. The abdominal cavity after surgery was examined without any pathological findings.

All pigs had a well healed suture without any secretion in the* linea alba*. In the abdominal cavity, a fibrous synechia was detected in parts of the resected liver lobes with peritoneum and some adhesions were found between the liver lobes and stomach, omentum, or loops of jejunum. Resection margins in the liver lobes were mostly covered with fibrous tissue at the adhesions. Four female pigs had a less serous effusion in their abdominal cavities. The thoracic cavity in four pigs was without adhesions and effusions. The lungs of five pigs had pathological findings consisting of atelectatic bearings—acute inflammatory or chronic bronchopneumonia. A fibrinoid, purulent pericardial effusion was observed in one of these pigs. Samples of the right and left hepatic lobe were collected from all pigs for a histological examination.

The histopathological evaluation of thermal changes at the resection margin showed a strong thermal alteration in both groups. The depth of the thermal alteration was on average 4 mm larger in group A using the newly tested instrument; dispersion of values was 12–17 mm. Statistical analysis using the median test (*P* value was computed by Fisher's exact test) did not show any significant differences between the groups (Table 3).

The statistical analysis showed that there were not any differences between the tested group A and the standard group B.

## 4. Discussion

**Figure 7:**
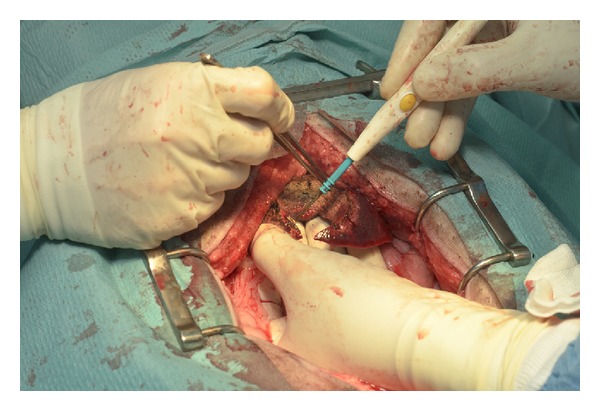
*In vivo* study—operating field and condition of liver tissue after semispherical excision.

Our* ex vivo* and* in vivo* experimental studies demonstrated the feasibility and safety of the newly developed semispherical bipolar device RONJA for the liver ablation and resection. There were no statistically significant differences between the new tested device and the generally used multiple needle device. A spherical area of coagulated necrosis was achieved in all cases using the new semispherical radiofrequency device RONJA (Figure 7).

The main advantages of the RONJA device against the commonly used multiple needle device include reduction of needle punctures into liver tissue, faster ablation time, and, therefore, reduction of total operating time and of possibility of repeated resections in case of metastatic relapse or progression of the disease because of a better preservation of the healthy liver tissue.

**Figure 8:**
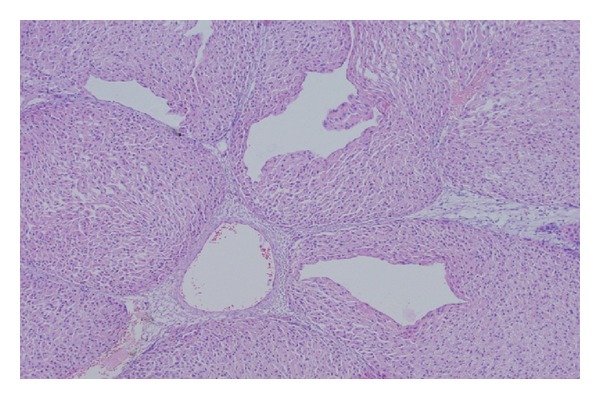
Microscopic section of liver biopsy, thermal alteration of hepatic tissue, perivasal edema, and central veins dilatation.

Many authors have used a porcine model in feasibility and safety studies of devices for radiofrequency ablation. Wright et al. compared radiofrequency and microwave ablation on the group of nineteen pigs [9]. They concluded that microwave and radiofrequency ablation zones are similar in pathologic appearance and imaging characteristics. Using our RONJA device, we also have not observed any difference in the histopathological evaluation of the ablation (Figure 8).

Varadarajulu et al. (2009) performed RFA of porcine liver using the retractable umbrella-shaped electrode array with effective coagulation necrosis of large areas [10]. Lee et al. (2013) confirmed that RFA procedures using 15-G Octopus electrodes are useful and safe for creating a large ablation of porcine liver in a single electrode model as well as in the multiple electrodes model [11].

Schutt et al. used a porcine model with the aim of testing a new device consisting of a linear array of blade-shaped 1 cm wide radiofrequency (RF) electrodes 1.5 cm apart. A total of 7 liver lobes were resected from 2 domestic swine in this study. The authors developed a device that rapidly coagulated the hepatic resection plane in an animal model. In the authors' opinion the new device shows promise in the effort to reduce blood loss and operating time during liver and kidney resections [12].

## 5. Conclusion

We have designed and developed a new semispherical bipolar radiofrequency device that rapidly coagulates the hepatic resection plane in a porcine model. Our prototype was able to coagulate the semispherical contour of liver parenchyma with successful coagulation of all blood vessels. This device shows promise in efforts to reduce healthy liver parenchyma resection during liver surgery in a porcine model. By performing resection of pig liver parenchyma using the new semispherical device, we have confirmed that this new method is effective and safe. The device could be used in the treatment of small malignancies (up to 2 cm in diameter) in peripheral parts of liver lobes. However, it is not suitable for resections in the area of hepatic hilus.

Future animal and clinical studies will need to evaluate semispherical liver RF resection with respect to the local recurrence of hepatic tumors.

## Figures and Tables

**Table 1:** Operating data: there was a significant difference (*P* = 0.005) in supplied power between the groups.

	Standard device;	RONJA;	*P* value
	mean ± S.E.M. (median)	mean ± S.E.M. (median)	
Weight 1 (kg)	50.83 ± 3.55 (47.50)	53.00 ± 4.69 (55.50)	0.567
Total operating time (min)	69.00 ± 6.76 (77.00)	57.50 ± 3.99 (53.50)	0.567
Ablation time of left lobe (min)	11.67 ± 2.11 (11.50)	8.83 ± 1.25 (7.50)	0.567
Ablation time of right lobe (min)	8.50 ± 0.89 (8.00)	7.33 ± 0.84 (7.00)	1
Supplied power (W)	90.00 (unchanging)	81.67 ± 1.67 (80.00)	**0.005**
Weight 2 (kg)	52.92 ± 3.85 (49.50)	55.17 ± 4.11 (57.50)	0.567
Laparoscopic total time (min)	25.33 ± 5.08 (23.50)	27.17 ± 4.66 (23.00)	1
Actual time of laparoscopy (min)	13.83 ± 3.89 (14.00)	6.00 ± 1.16 (5.00)	0.567
Weight 3 (kg)	58.82 ± 4.35 (61.00)	58.83 ± 4.89 (59.90)	1
Total weight increment (kg)	6.62 ± 1.46 (7.00)	5.83 ± 0.88 (6.65)	0.567
Liver weight (kg)	1.54 ± 0.13 (1.50)	1.48 ± 0.11 (1.48)	1

**Table 2:** Data concerning complications: there were no significant differences between the groups.

	Standard device; *n* (%)	RONJA; *n* (%)	*P* value
Complications during surgery	0 (0)	1 (16.7)	1
Postoperative complications	1 (16.7)	0 (0)	1
Complications during laparoscopy	0 (0)	4 (66.7)	0.061
Postoperative adhesions (liver)	6 (100)	6 (100)	—
Postoperative adhesions (spleen)	2 (33.3)	2 (33.3)	1
Pathological findings—ascites	1 (16.7)	2 (33.3)	1
Pathological findings—abscess	0 (0)	1 (16.7)	1
Pathological findings in lungs	4 (66.7)	2 (33.3)	0.567

**Table 3:** Data concerning thermal alteration: there were no significant differences between the groups.

	Standard device;	RONJA;	*P* value
	mean ± S.E.M. (median)	mean ± S.E.M. (median)	
Thermal alteration at the resection margin (%)—right lobe	98.33 ± 1.67 (100.00)	98.33 ± 1.67 (100.00)	—
Thermal alteration at the resection margin (%)—left lobe	100.00 (unchanging)	95 ± 3.42 (100.00)	—
Maximum depth of necrosis (mm)—right lobe	13.00 ± 1.61 (13.00)	14.83 ± 2.20 (14.00)	1
Maximum depth of necrosis (mm)—left lobe	13.67 ± 1.94 (14.00)	14.00 ± 0.97 (13.50)	1
Weight of resected particle (g)—right lobe	9.55 ± 1.37 (10.20)	15.33 ± 2.65 (14.40)	0.567
Weight of resected particle (g)—left lobe	11.75 ± 1.29 (11.30)	14.75 ± 1.05 (14.25)	0.567
Maximum depth of thermal alteration (cm)—right lobe	1.08 ± 0.08 (1.00)	1.42 ± 0.07 (1.40)	0.08
Maximum depth of thermal alteration (cm)—left lobe	1.08 ± 0.05 (1.00)	1.45 ± 0.07 (1.45)	0.08
